# Carbon monoxide-releasing molecule CORM-401 treatment elicits corticosterone-driven stress lipolysis and tissue-specific hypoxia-inducible factor activation

**DOI:** 10.1016/j.redox.2026.104295

**Published:** 2026-07-10

**Authors:** Emma Klemola, Karoliina Posio, Mikko Karpale, Elitsa Y. Dimova, Ghulam S. Raza, Kari A. Mäkelä, Irina I. Nagy, Ilkka Miinalainen, Joona Tapio, Peppi Koivunen

**Affiliations:** a Research Unit of ECM & Hypoxia, Faculty of Biochemistry and Molecular Medicine, University of Oulu, Finland; b Biocenter Oulu, University of Oulu, Finland; c Research Unit of Biomedicine and Internal Medicine, Faculty of Medicine, University of Oulu, Oulu, Finland; d Department of Clinical Chemistry, Research Unit of Cancer and Translational Medicine, Medical Research Center, University of Oulu and Northern Finland Laboratory Centre NordLab, Oulu University Hospital, Oulu, Finland

**Keywords:** Carbon monoxide, Carboxy hemoglobin, Hemoglobin, HIF, Hypoxia, Metabolic syndrome

## Abstract

Metabolic syndrome is a global health concern characterized by obesity, insulin resistance, dyslipidemia, and hypertension – all of which increase risk of cardiovascular diseases and type 2 diabetes. CO-releasing molecules (CORMs) deliver low amounts of CO *in vivo* and have been reported to improve metabolic parameters in obese mice by inducing a transient mitochondrial uncoupling and improving insulin resistance. CO reduces oxygen-binding capacity of hemoglobin, which may cause tissue hypoxia and mediate metabolic alterations through the hypoxia-inducible factor (HIF) pathway. This study: 1) Analyzes whether the beneficial metabolic effects of CORMs are mediated by the HIF pathway, and 2) Evaluates the metabolic effects of long-term CORM-401 treatment in high-fat diet-fed mice. A 7-week-treatment of CORM-401 elicited a metabolic phenotype characterized by significantly reduced body weight and white adipose tissue (WAT) mass, increased energy expenditure and glucose tolerance, and higher LDL + VLDL cholesterol levels. CORM-treatment triggered lactatemia-induced metabolic acidosis which was compensated through increased respiration. No toxicity or organ damage was seen. HIF target mRNA levels were positively associated with carboxyhemoglobin levels in the CORM-401-treated tissues. CORM-401-treated mice exhibited elevated serum corticosterone levels, which showed associations with metabolic mRNAs in WAT and liver. These findings suggest a dual mechanism: glucocorticoid-driven stress activation as the primary mechanism accompanied by a low-grade, tissue specific HIF engagement underlying the observed metabolic effects of the CORM-401 treatment. Despite the mild beneficial effects on metabolism, the systemic hormonal effects of the long-term CORM-401 treatment warrant caution when evaluating its potential as a therapeutic for metabolic disorders.

## Highlights

• Prolonged CORM-401 causes weight loss mainly via reduced WAT mass.
• CORM-401 boosts locomotor activity, water intake, and energy expenditure.
• CORM-401 raises serum LDL + VLDL and induces hepatic cholesterol synthesis mRNAs.
• Prolonged CORM-401 elevates circulating corticosterone levels.
• Long-term CORM-401 mildly activates the HIF pathway in metabolic WAT, liver and skeletal muscle.

## Graphical abstract

**Figure:**
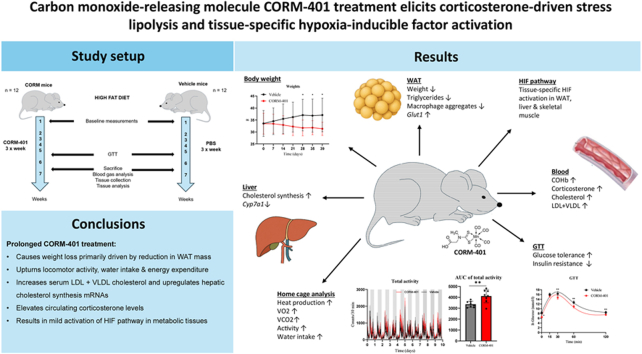

## 1 Introduction

Metabolic syndrome (MetS) is a cluster of conditions that increases the risk for developing cardiovascular diseases (CVDs), type 2 diabetes (T2D) and chronic kidney disease (CKD) [1]. The global spread of “Western lifestyle” has caused MetS to become a pandemic and a major health hazard of the modern world, currently estimated to affect a quarter of the worlds’ population [1,2]. CVDs and T2D account for approximately 35% of global total mortality [3,4]. A lower prevalence of obesity, T2D, ischemic heart disease, and related mortality has been reported among people living at high altitude, thus exposed to lower ambient oxygen concentrations [5].

Insufficient oxygen availability is primarily sensed at cellular level via the hypoxia-inducible factor (HIF) pathway. HIF is a heterodimeric protein composed of a stable β subunit and an α subunit that is sensitive to oxygen-dependent degradation via prolyl 4-hydroxylases (HIF–P4Hs 1–3, aslo knows as PHDs and EGLNs). Under hypoxic conditions, inhibition of hydroxylation enables formation of the HIF αβ heterodimer, which activates transcription of over 300 genes involved in metabolism, erythropoiesis, angiogenesis, and inflammation [5].

Small-molecule HIF–P4H inhibitors are already in clinical use for anemia in chronic kidney disease (CKD) [[6], [7], [8]]. Because HIF regulates many pathways, inhibition of HIF–P4Hs has been widely studied. Detailed animal model studies show that mice hypomorphic for HIF–P4H-2 are leaner, show improved glucose and lipid metabolism, and are protected from atherosclerosis and fatty liver disease [[9], [10], [11]]. Similar benefits occur when HIF–P4H-2 is silenced specifically in adipose tissue [12].

Hemoglobin (Hb) is an iron-containing metalloprotein in red blood cells and the main carrier of O_2_ in the body. Hb levels are regulated both genetically and environmentally by sex, race, age, altitude, and smoking status; male sex, higher living altitude and smoking elevating Hb levels [13]. Lower-end Hb levels within normal variation are associated with better glucose tolerance, lower cholesterol and blood pressure (BP) levels, less adverse metabolite profiles and a lower inflammatory load, and the lower Hb levels associate with activation of the HIF-response [[14], [15], [16]]. Moreover, higher Hb levels are associated with a greater risk of developing gestational diabetes and both CVD-related and total mortality [17,18].

Carbon monoxide (CO) is formed endogenously in mammals during hemoprotein catabolism, when Hb is broken down [19]. CO has approximately 220 times stronger affinity to Hb than O_2_ leading to formation of carboxyhemoglobin (COHb), and decreased levels of oxyhemoglobin (O_2_Hb) and tissue oxygenation. While in large concentrations COHb is highly toxic to humans [20], at low concentrations, however, CO mediates several biological processes including vasorelaxation, inflammation, mitochondrial functions, cell proliferation, and apoptosis [21]. CO-releasing molecules (CORMs) are orally administered compounds capable of releasing low amounts of CO *in vivo* [22,23]. Previous animal experiments have shown that CORM-401 improves insulin resistance and reduces adiposity, and adipose tissue inflammation in obese mice [24,25]. CORM-molecules have also been investigated in context beyond metabolic regulation for example in sickle cell disease, prostate cancer and renal dysfunction [[26], [27], [28], [29]]. According to current understanding, CORMs transport CO into tissues and cause an uncoupling of mitochondrial respiration, leading to transient decrease in mitochondrial ATP production and a metabolic switch toward glycolysis [24].

As both hypoxia/HIF signaling and CO biology have been linked to the key aspects of metabolic dysfunction, the aims of this study were to: 1) Analyze whether the effects of CORM-401 involved the HIF pathway, and 2) Evaluate the effects of long-term CORM-401 treatment on glucose and lipid metabolism in high-fat diet fed mice.

## 2 Methods

### 2.1 Kinetic activity assays

Recombinant human HIF–P4H-1, HIF–P4H-2, and HIF–P4H-3 were expressed in insect cells by baculoviruses, affinity purified using anti-Flag, and their catalytic activity was measured using a previously reported method based on the hydroxylation-coupled stoichiometric release of ^14^CO_2_ from 2-oxo [1-^14^C]glutarate [30].

IC50 values for CORM-401 were determined by varying its concentration while keeping concentrations of other cofactors and cosubstrates constant. Values were calculated from saturation curves in Excel (Microsoft) as the average of at least three independent assays.

### 2.2 Preparation of CORM-401, backbone, and vehicle

CORM-401 (MedChemExpress, USA) was diluted in sterile PBS without Ca^2+^ and Mg^2+^ (Dulbecco's Phosphate Buffered Saline, USA), which was used as the vehicle to prepare a 15 mM stock solution. Drops of 1 M NaOH were added and the dilution was vortexed thoroughly and sonicated for 20-30 min until the solution was clear yellow. As another negative control, MnSO4 (Sigma Aldrich) and sarcosine (Sigma Aldrich) were diluted in sterile PBS (termed backbone) to match their concentration in CORM-401 enabling study of the effects of Mn^2+^ and ligand backbone without CO release. Mice were weighed at baseline and weekly after starting the CORM-401 or vehicle treatment. According to the weekly weights, the 30 mg/kg doses were prepared for each mouse by diluting the stock solution in sterile PBS.

### 2.3 Animal experiments

Wild-type C57BL6/J male mice were used as experimental animals in all the experiments. The animal experiments were performed in accordance with protocols approved by the National Animal Experiment Board of Finland (ESAVI/31050/2024). The mice in all experiments were housed individually in the Laboratory Animal Center of the University of Oulu with a temperature of 21 to 22 °C and 12-h day/night cycle. Well-being of the mice was monitored daily by animal caretakers.

#### 2.3.1 Pilot experiment

A single-dose pilot experiment was performed prior to the main experiment. 3-month-old mice fed normal chow received a single dose of CORM-401 (30 mg/kg) or its chemical backbone (C_3_H_6_NO_2_S_2_) by oral gavage, and were sacrificed at three different time points (0 min, 5 min, or 2 h following the gavage). At sacrifice venous blood samples were taken under terminal anesthesia and blood gas analysis was performed. WAT and liver tissues were snap frozen for HIF1α Western blotting and qPCR analysis of HIF target mRNAs.

#### 2.3.2 Main experiment

In the main experiment, 4-month-old mice were put on a high-fat diet (HFD) (C 1090 – 60, Experimental Diets), which has 60% of energy from fats, 6 weeks before the start of the treatment. Mice were orally administered either CORM-401 (30 mg/kg) or vehicle (PBS) 3 times a week (Monday, Wednesday and Friday before noon) for 7 weeks. A glucose tolerance test (GTT) was performed after 5 weeks of the treatment. At sacrifice blood samples were taken from mice under terminal anesthesia and tissues were collected for subsequent comprehensive analyses.

#### 2.3.3 Home cage analysis

In the home cage analysis, the study setup and experimental conditions were the same as of the main experiment. After 5 weeks of CORM-401 or vehicle treatment the mice were transferred to the animal monitoring room where the home cage analysis resides and placed in individual training cages for 7 days. After the training period, the mice were placed for 10 days in Phenomaster monitoring cages (TSE Systems GmbH, Berlin, Germany) which measure *ad libitum* drinking and feeding behavior, home cage activity and breathing gases. CORM-401 and vehicle treatments were contunued during the training and monitoring periods.

### 2.4 Collection of mouse tissues

All baseline blood and serum samples were obtained from the *vena saphena.* Fasting samples were measured after a 12-h fast, blood (b) Hb levels were measured using a Hb meter (HemoCue Hb 201+) and b-glucose levels with a glucometer (Contour, Bayer). B-Lactate was measured using a lactometer (SensLab/EKF Diagnostics). Serum was separated out by centrifugation at 2000 x *g* for 20 min at 4 °C. At sacrifice non-fasted blood samples were taken from inferior *vena cava* (IVC) under terminal anesthesia of fentanyl-medetomidine-midazolam (FMM). Body weight, gonadal white adipose tissue (WAT), brown adipose tissue (BAT) and liver weights were measured at sacrifice. Tissues were either snap-frozen in liquid nitrogen or fixed in formalin (4% formaldehyde, VWR) overnight for histological analysis. Weight change during the experiment was determined with the formula “final weight – baseline weight”.

### 2.5 Chemistry analysis of mouse blood and tissue samples

Blood gas analysis was performed using an ABL90 blood gas analyzer (Nordlab, Oulu University of Hospital). Samples were collected at the minimum volume required for PICO 70 syringes (0.3–0.5 mL), following validated preanalytical and analytical protocols for hospital samples with a minimal delay; maximum time to test of 40 min. Serum (s) insulin levels were determined with a Rat/Mouse Insulin ELISA kit (EZRMI-13 K, Millipore) and s-total cholesterol, s-HDL cholesterol, s-triglyceride and liver and WAT triglyceride levels by enzymatic methods (Roche Diagnostics). S-free fatty acid (FFA) levels were determined with Free Fatty Acid Quantification Assay Kit (Abcam). Glycogen concentrations were determined using Glycogen Assay kit (Cayman Chemicals) of ∼100 mg snap-frozen liver and ∼60 mg skeletal muscle. S-albumin (ALB), s-urea, s-urate, s-alanine aminotransferase (ALT), s-alkaline phosphatase (ALP) and s-creatinine (Cr) concentrations were measured with Siemens ADVIA Chemistry XPT analyzer following validated preanalytical and analytical protocols for hospital samples (Nordlab, Oulu University Hospital). S-corticosterone levels were determined using ELISA kit (Arbor Assays). Serum beta-hydroxybutyrate concentration was measured using Beta-Hydroxybutyrate Assay Kit (Sigma Aldrich). Liver cholic acid concentrations were measured using liquid chromatography-mass spectrometry (LC-MS) analysis. ∼100 mg snap-frozen liver tissues were homogenized in 300 μl of 50% Methanol (MeOH) (1 + 3, 4-fold) for LC-MS analysis (Thermo Scientific™ Q Exactive™ Plus Quadrupole-Orbitrap™ Mass Spectrometer with Waters Acquity UPLC system, Proteomics and Protein Analysis Core, Biocenter Oulu, Finland).

### 2.6 Histological and immunohistochemical analyses

Formalin-fixed tissues were embedded in paraffin, cut into 5 μm thin sections and stained with hematoxylin and eosin (H&E). The slides were imaged using a Hamamatsu NanoZoomer S60 slide scanner. The level of liver steatosis was manually assessed based on the quantity and size of lipid droplets, using a scale of “mild”, “moderate”, or “severe”. WAT macrophage aggregates were visualized using CD68 immunostaining (Abcam, lot GR125743-1) and the number of aggregates in a 1 × 1 mm area of the tissue were calculated. A semi-quantitative scoring scale was established where “mild” corresponded to 0-1 aggregates per 1 × 1 mm area, “moderate” to 2-3 per 1 × 1 mm area, and “severe” to 4 or more aggregates per 1 x 1 mm. WAT browning was assessed by staining with an anti-UCP1 antibody (Sigma-Aldrich, lot 035M4823V) using 1:500 dilution. The number of renal glomeruli was determined manually by randomly selecting 1 × 1 mm regions of the HE-stained kidney sections and counting all glomeruli within each region.

### 2.7 Transmission electron microscope (TEM) analyses

At sacrifice, small pieces of WAT, BAT and liver tissues were cut into tiny sections and further fixed, and post-fixed in 1% osmium tetroxide, dehydrated in acetone, and embedded in Epon LX112. Thin sections were cut with a Leica Ultracut UCT ultramicrotome and examined in Tecnai G2 Spirit transmission electron microscope. The images were taken using a Quemesa CCD camera (Olympus Soft Imaging Solutions) and mitochondria were manually assessed in each sample.

### 2.8 mRNA extraction and qPCR analyses

mRNA was extracted from liver and skeletal muscle samples using the TriPure Isolation Reagent (Roche Applied Science), followed by purification with the E.Z.N.A. Total RNA Kit I (Omega Bio-Tek). For WAT and BAT samples, RNA purification was performed with the E.Z.N.A. Total RNA Kit II (Omega Bio-Tek). mRNA was transcribed into cDNA using the iScript cDNA synthesis kit (Bio-Rad) using 500 ng of mRNA as a template. Quantitative PCR was performed with iTaq SYBR Green Supermix with ROX (Bio-Rad) in a C1000 Touch Thermal Cycler and a CFX96 Touch Real-Time PCR Detection System (Bio-Rad) with the primers shown in Table 1.

**Table 1:** Primer sequences used in RT-qPCR.

Gene	Forward primer	Reverse primer
*Acsl1*	TGCCAGAGCTGATTGACATTC	GGCATACCAGAAGGTGGTGAG
*Bact*	CGATGCCCTGAGGCTCTTTTC	TCTTTACGGATGTCAACGTCACACT
*B2m*	GGTCTTTCTGGTGCTTGTCTCA	GTTCGGCTTCCCATTCTCC
*Ccl2*	CCTGCTGTTCACAGTTGCC	ATTGGGATCATCTTGCTGGT
*Cebpa*	CAACCTGGAGACGCAGCACAAG	GCTTGAACAAGTTCCGCAGGGT
*Cidea*	TGACATTCATGGGATTGCAGAC	GGCCAGTTGTGATGACTAAGAC
*Cox1*	TAACTTCCTCACTCGAAGCCA	AGTTCCATGACCCATCTCTGTC
*Cyp51*	GGAGCGAAAAGTCCACCAC	GCATCACTCCCCAGAAGGTA
*CYP7A1*	TTTCCATCACTTGGGTCTATGC	AAACTCCCTGTCATACCACAAAG
*CYP8B1*	GCACCGTGAAGACATCCCC	CCTCTGGACAAGGGTTTTGTG
*Fdps*	GGAGGTCCTAGAGTACAATGCC	AAGCCTGGAGCAGTTCTACAC
*Glut1*	TCAAACATGGAACCACCGCTA	AAGAGGCCGACAGAGAAGGAA
*Glut4*	ACACTGGTCCTAGCTGTATTCT	CCAGCCACGTTGCATTGTA
*Hmgcr*	AGAGCGAGTGCATTAGCAAAG	GATTGCCATTCCACGAGCTA
*Ldha*	GCATGAGCTTGCCCTTGTTGA	GACCAGCTTGGAGTTCGCAGTTA
*Lep*	GAGACCCCTGTGTCGGTTC	CTGCGTGTGTGAAATGTCATTG
*Lipe*	CAGAAGGCACTAGGCGTGATG	GGGCTTGCGTCCACTTAGTTC
*Lipin1*	GCTCCCGAGAGAAAGTGGTGGA	GGCTTTCCATTCTCGCAGCTCCT
*Pdk1*	AGGATCAGAAACCGGCACAAT	GTGCTGGTTGAGTAGCATTCTAA
*Ppara*	AGAGCCCCATCTGTCCTCTC	ACTGGTAGTCTGCAAAACCAAA
*Pargc1a*	AGCCGTGACCACTGACAACGAG	GCTGCATGGTTCTGAGTGCTAAG
*Pparg2*	TCGCTGATGCACTGCCTATG	GAGAGGTCCACAGAGCTGATT
*Ppia*	GAGCTGTTTGCAGACAAAGTTC	CCCTGGCACATGAATCCTGG
*Prdm16*	CCACCAGCGAGGACTTCAC	GGAGGACTCTCGTAGCTCGAA
*Srebp2*	TGGGCGATGAGCTGACTCT	CAAATCAGGGAACTCTCCCAC
*Tbp*	AGAACAATCCAGACTAGCAGCA	GGGAACTTCACATCACAGCTC
*Ucp1*	AGGCTTCCAGTACCATTAGGT	CTGAGTGAGGCAAAGCTGATTT
*Vegfa*	GCACTGGACCCTGGCTTTAC	AACTTGATCACTTCATGGGACTTCT

### 2.9 Western blotting

Nuclear and cytosolic fractions were extracted from ∼100 mg snap-frozen liver, ∼150 mg WAT and ∼50 mg skeletal muscle using the NE-PER kit (ThermoFisher Scientific). Nuclear fractions (40 μg) were resolved by 10% SDS-PAGE, blotted (Immobilon-P membrane, Millipore), and probed with primary antibodies against HIF1α (NB100-479, Novus Biologicals, 1:500 or D1S7W XP® Rabbit mAb, Cell Signaling Technologies) and β-actin (NB600-501, clone AC-15, Novus Biologicals, 1:5000). The secondary antibody, either anti-mouse or anti-rabbit, was conjugated to horseradish peroxidase (DAKO, P0447 and P0448, 1:5000). PageBlue (ThermoFisher Scientific) staining was used for detection of proteins on the membrane. RCC cell lysate was used as a positive control for HIF1α. For detection the Clarity Max Western ECL Substrate (Bio-Rad) and Pierce ECL Western Blotting Substrate (Thermo Fisher Scientific) were used.

### 2.10 Glucose tolerance test (GTT)

GTT was performed without anesthesia after treating mice for 5 weeks with the CORM-401 or vehicle. The mice were fasted 12 h prior to GTT. Glucose (1 mg/g) was administered via an i.p. injection. B-glucose was measured from the lateral tail veins at baseline and 15, 30, 60 and 120 min. Area under curve (AUC) for GTT was calculated by the summary measures method, and the homeostatic model assessment of insulin resistance (HOMA-IR) scores from the fasting b-glucose and fasting s-insulin values were calculated by a formula = (insulin (pmol/l)∗b-glucose (mmol/l))/22.5.

### 2.11 Cellular experiments with primary mouse hepatocytes

Primary mouse hepatocytes were isolated using a two-step perfusion method. A five-week-old C57BL/6 J mice was euthanized by cervical dislocation. IVC was cannulated, the portal vein was incised, and the liver was perfused with prewarmed (+37 °C) perfusion medium (Thermo Fisher, 17701038) at a flow rate of 1 ml/min. After 5 min, the perfusion medium was replaced with liver digest medium (Thermo Fisher, 17703034), and a total volume of 11 ml was perfused through the liver. Following digestion, the liver was excised and transferred to a Petri dish containing plating medium (William's E medium (Sigma, W1878) supplemented with 0.1 μM dexamethasone and thawing and plating supplements (Thermo Fisher, CM3000). The liver capsule was gently disrupted using forceps to release hepatocytes into the medium. The resulting cell suspension was filtered through a 40 μm cell strainer into a Falcon tube and centrifuged at 50 × g for 5 min at 4 °C. The supernatant was discarded, and the cell pellet was resuspended in plating medium. Cell number and viability were determined using Trypan blue staining and a hemocytometer. Cells were seeded at a density of 3.5 × 10^5^ cells per well in 24-well plates. After 4 h, the medium was replaced with serum-free maintenance medium (Sigma, William's E medium, W1878) supplemented with 0.1 μM dexamethasone and cell maintenance cocktail (Thermo Fisher, CM4000). Treatments with 1 μM CORM-401 or vehicle control (0.1% DMSO) were initiated the following day. RNA samples were collected after 24 h of treatment.

### 2.12 Statistical analyses

Student's homoscedastic two-tailed *t*-test was used to compare two groups and two-tailed paired *t*-test to compare two data points within the group. Data are presented as means ± SD unless otherwise stated. Coefficient of determination (R^2) was used to determine the associations between two continuous variables. Fisher's exact test was used to compare categorized data from histological analyses. *p* < 0.05 was considered statistically significant. In the figures statistical significance is indicated by asterisks: ∗ = *p* < 0.05, ∗∗ = *p* < 0.01, ∗∗∗ = *p* < 0.001, ∗∗∗∗ = *p* < 0.0001. Figures were prepared using GraphPad Prism 10.2.2 software.

## 3 Results

### 3.1 CORM-401 inhibits HIF–P4Hs 1-3 concentration dependently and its single administration to mice increases COHb levels but does not show robust activation of the HIF response in tissues

**Fig. 1:**
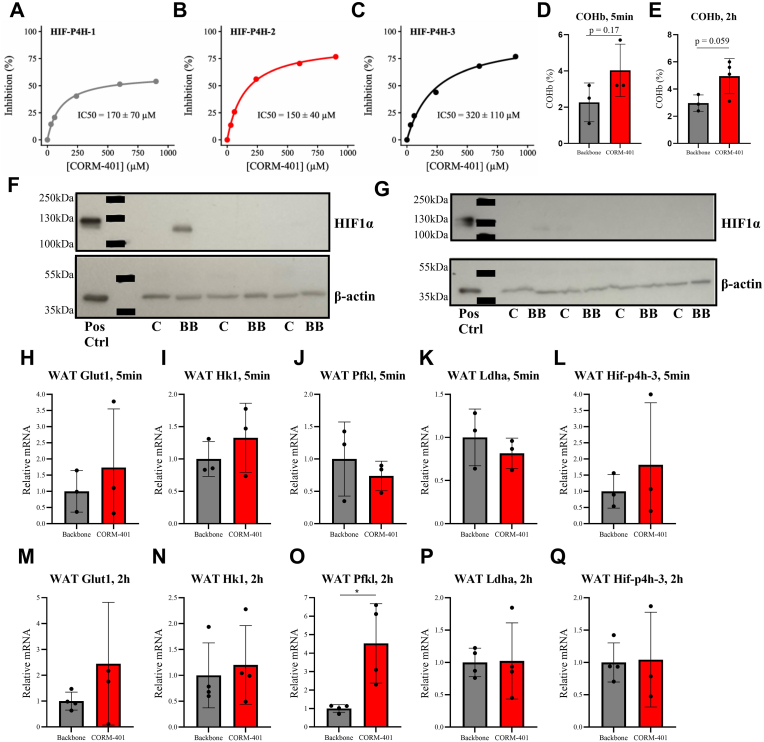
Enzyme inhibitory assays and a single-dose pilot experiment of CORM-401. A-C, enzyme inhibitory measurements of CORM-401 with human HIF–P4Hs 1-3. D-E, blood carboxyhemoglobin (COHb) concentration of backbone (*n* = 3) and CORM-401-administered mice (*n* = 3-4) at 5 min and 2 h timepoint. F-G, HIF1α Western blot of WAT at 5 min and 2 h timepoint, respectively. H-Q, qPCR analysis of WAT mRNA levels of CORM-401 administered mice (*n* = 3-4) relative to backbone (*n* = 3-4) at 5 min and 2 h timepoint, studied relative to *Ppia* mRNA. C, CORM-401; BB, backbone; Pos Ctr, positive control. *Glut1*, glucose transporter 1; *Hk1*, hexokinase 1; *Pfkl,* phosphofructokinase liver type; *Ldha*, lactate dehydrogenase a; *Hif-p4h-3*, hypoxia-inducible factor prolyl 4-hydroxylase 3. ∗*p* < 0.05.

To investigate the connection between CORM-401 and HIF, we first performed enzyme inhibitory assays *in vitro*. These analyses showed that CORM-401 inhibited recombinant human full-length HIF–P4Hs 1-3 in a concentration-dependent manner, with IC50 values between 150 and 320 μM (Fig. 1A–C), whereas no such inhibition was seen with the molecular back bone (data not shown). A similar level inhibitory effect has previously been demonstrated with CORM-2 on the catalytic domain of HIF–P4H-2, supporting the notion that CO can directly modulate HIF–P4H enzyme activity [31].

To determine whether the effects of CORM-401 *in vivo* were mediated by released CO or the molecular backbone, we performed a single-dose pilot study where mice were administed 30 mg/kg of the compounds and sacrificed 0 min – 2 h after the gavage. COHb levels at 5 min and 2 h were 1.7-1.8x higher following CORM-401 than backbone administration, suggesting CO release, although the differences did not reach statistical significance likely due to the small number of mice in the pilot (Fig. 1D and E). The measured COHb levels were in line with an earlier report [24] and with the theoretical estimates of CO release.

We next examined potential effects at protein and gene expression levels. No HIF1α specific stabilization was detected in WAT or liver at either time point following CORM-401 administration (Fig. 1F and G, S1A and S1B). However, in a single backbone-administered mouse in WAT at 5 min, a HIF1α positive band was observed whereas the rest of the data did not support the conclusion of HIF1α stabilization (Fig. 1F and 1H-L). At the transcriptional level, a modest 1.3 - 1.7x upregulation of glucose transporter 1 (*Glut1, Slc2a1)*, hexokinase 1 (*Hk1)*, and *Hif-p4h-3* (*Egln3*) mRNA that did not reach significance was detected in WAT at 5 min (Fig. 1H–L). At 2 h, a statistically significant 4.5x upregulation of phosphofructokinase L (*Pfkl)* mRNA was observed, whereas the 2.3x upregulation in *Glut1* mRNA did not reach significance (Fig. 1M–Q). In the liver, no significant differences in gene expression were detected at either time point between the treatment groups (Fig. S1C–N).

### 3.2 Metabolic outcomes of the long-term CORM-401 treatment on obese mice

**Fig. 2:**
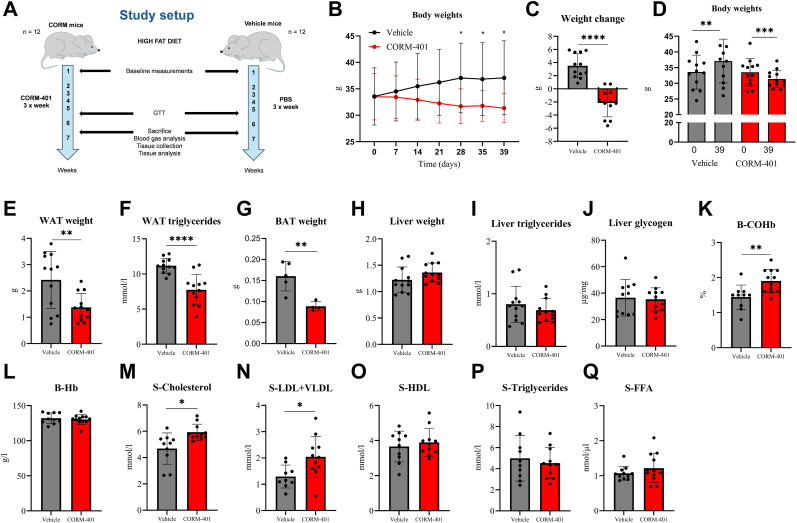
Long-term treatment of mice with CORM-401. *n* = 9-12 in each group. A, study setup. B, body weight development during the treatments. C, total weight change after 7 weeks of treatment. D, weights at day 0 and day 39. E, WAT weight. F, WAT triglyceride concentration. G, BAT weight. H, liver weight. I, liver triglyceride concentration. J, liver glycogen concentration. K, Carboxyhemoglobin (COHb) concentration 24 h after last treatment. L, Hb concentration. M, serum cholesterol concentration. N, serum low-density lipoprotein (LDL) and very low-density lipoprotein (VLDL) concentration. O, serum high-density lipoprotein (HDL) concentration. P, serum triglyceride concentration. Q, serum free fatty acid (FFA) concentration. ∗*p* < 0.05, ∗∗*p* < 0.01, ∗∗∗*p* < 0.001, and ∗∗∗∗*p* < 0.0001.

After the initial pilot experiment, the main experiment was conducted (Fig. 2A). Mice were first put on a HFD for 6 weeks which increased their body weight from 26.0 ± 2.0 g to 34.0 ± 5.0 g. HFD-feeding was continued, and the mice received three times a week 30 mg/kg CORM-401 or vehicle for 7 weeks; the final dose given 24 h before sacrifice (Fig. 2A). The mice were weighed weekly and after 5 weeks, a GTT was conducted (Fig. 2A). No difference between the study groups in body weight, blood Hb, glucose or lactate levels, or serum insulin or cholesterol levels were seen before start of the treatment (Fig. S2A–F). During the experiment, the vehicle-treated mice gained weight steadily whereas the CORM-401-treated mice gradually lost weight (Fig. 2B). This led to a marked, an almost 6 g difference in weight change between the study groups; the CORM-401-treated mice losing 2.2 g whereas the vehicle-treated gaining 3.5 g (Fig. 2C and D). This difference was primarily driven by a ∼45% reduction in WAT mass accompanied by a 30% reduction in WAT triglycerides (Fig. 2E and F), and also reflected in smaller adipocytes and significantly less WAT macrophage aggregates in the histological analyses in the CORM-401-treated mice compared to vehcile (Fig. S2G–I). BAT mass was also lower in the CORM-401-treated mice compared to vehicle (Fig. 2G). No difference in liver mass, liver triglyceride or glycogen concentration was observed between treatment groups but histological analyses showed less fat accumulation in the CORM-401-treated liver compared to vehcile (Fig. 2H–J and Fig. S2J–L). Blood COHb concentration 24 h after the last CORM-401 dose was slightly but significantly 1.2 x higher in the CORM-401-treated mice compared to vehicle, suggesting some CO build-up during the experiment (Fig. 2K), no difference in blood Hb levels being observed (Fig. 2L). The CORM-401-treated mice had higher serum total cholesterol levels (Fig. 2M), driven by higher LDL + VLDL cholesterol levels (Fig. 2N) while no difference in HDL cholesterol, triglyceride or FFA levels being observed (Fig. 2O–Q).

### 3.3 CORM-401 improves glucose tolerance and reduces insulin levels

**Fig. 3:**
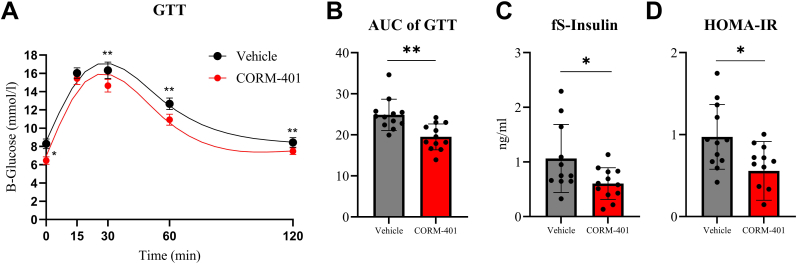
Glucose tolerance test. A, glucose tolerance test (GTT). *n* = 12 in each group. The value for 0 min was determined after 12 h fasting. B, area under curve (AUC) of GTT. C, fasting insulin levels. D, homeostatic model assessment for insulin resistance (HOMA-IR). ∗*p* < 0.05, ∗∗*p* < 0.01.

After 5 weeks of the treatment, a GTT was performed. The CORM-401-treated mice had a better glucose tolerance compared to vehicle-treated mice, with reductions observed in both fasting and 2 h glucose levels (Fig. 3A) and in the AUC of GTT (Fig. 3B). The CORM-401-treated mice had lower fasting insulin levels (Fig. 3C) and lower insulin resistance scores (HOMA-IR) compared to vehicle mice, suggesting better insulin sensitivity (Fig. 3D).

### 3.4 Higher COHb levels at sacrifice are associated with upregulation of HIF target genes

**Fig. 4:**
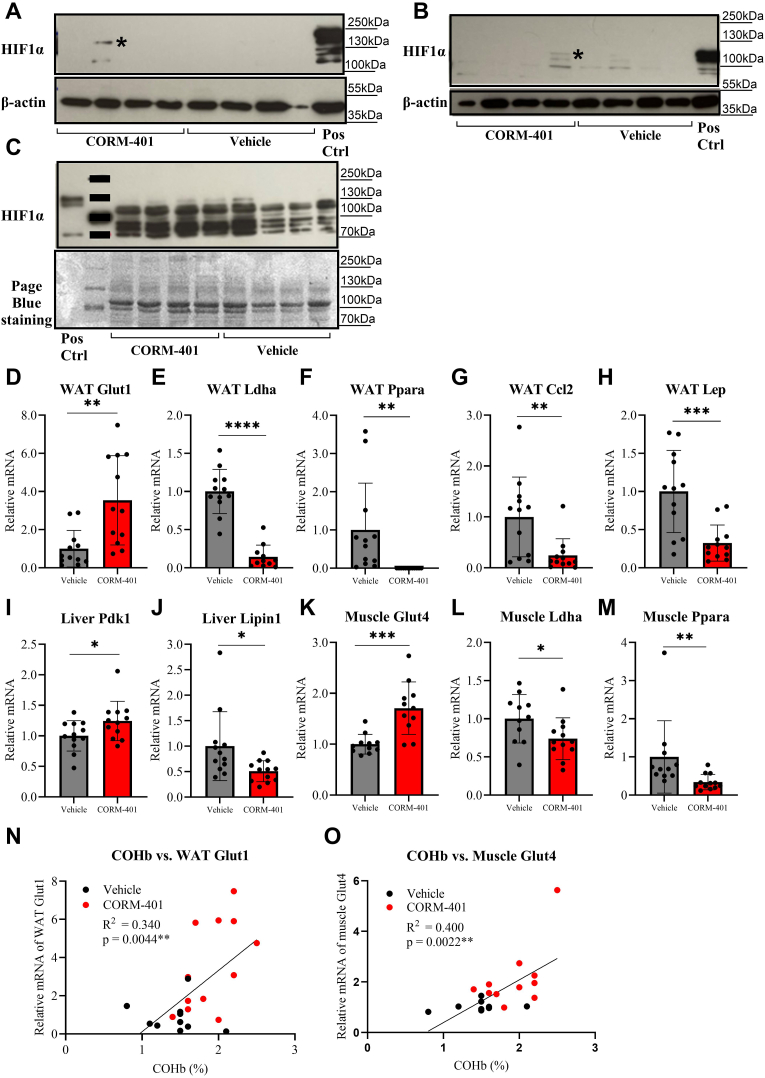
HIF target genes were upregulated concominantly with elevated COHb levels in CORM-401-treated tissues. *n* = 10-12 in each group. A-C, HIF1α Western blot of WAT, liver, and skeletal muscle, respectively. β-actin was used as a loading control for A and B, and PageBlue staining for C. D-H, qPCR analysis of WAT mRNA levels of CORM-401-treated mice relative to vehicle, studied relative to *Ppia* mRNA. I and J, qPCR analysis of liver mRNA levels of CORM-401-treated mice relative to vehicle, studied relative to *B2m* mRNA. K-M, qPCR analysis of muscle mRNA levels of CORM-401-treated mice relative to vehicle, studied relative to *bactin* mRNA. N and O, associations between blood COHb and WAT *Glut1* relative mRNA levels and blood COHb and skeletal muscle *Glut4* relative mRNA levels, respectively. ∗Stabilized HIF1α in A-B. *Ppara*, peroxisome proliferator-activated receptor a; *Glut1*, glucose transporter 1; *Ldha*, lactate dehydrogenase a; *Ccl2*, chemokine ligand 2; *Lep*, leptin; *Pdk1*, pyruvate dehydrogenase kinase; *Glut4*, glucose transporter 4. ∗*p* < 0.05, ∗∗*p* < 0.01, ∗∗∗*p* < 0.001, and ∗∗∗∗*p* < 0.0001.

To evaluate whether the long-term CORM-401 administration influenced hypoxia signaling, we next analyzed HIF1α protein levels and the expression levels of the key metabolic HIF target mRNAs in the WAT, liver, and skeletal muscle of the mice treated for 7 weeks by CORM-401 or vehicle. Weak stabilization of HIF1α was detected in one out of four CORM-401-treated WAT and liver but not in skeletal muscle (Fig. 4A–C). The CORM-401-treated mice showed upregulation of *Glut1* mRNA but downregulation of lactate dehydrogenase (*Ldha*), peroxisome proliferator-activated receptor α (*Ppara*), chemokine ligand 2 (*Ccl2*) and leptin (*Lep*) mRNA in WAT compared to vehicle (Fig. 4D–H). In liver, the CORM-401-treated mice showed upregulation of pyruvate dehydrogenase kinase 1 (*Pdk1*) mRNA but downregulation of lipin 1 (*Lipin1*) mRNA compared to vehicle (Fig. 4I and J). In muscle, the CORM-401-treated mice showed upregulation of *Glut4* (*Slc2a4*) mRNA but downregulation of *Ldha* and *Ppara* mRNA compared to vehicle (Fig. 4K–M). When the HIF target gene relative mRNA levels were blotted against blood COHb levels in all animals, significant positive associations were seen between COHb and WAT *Glut1* mRNA and COHb and skeletal muscle *Glut4* mRNA (Fig. 4N and O).

### 3.5 CORM-401-treated mice drink more water, are more active, produce more heat, consume more O_2_ and produce more CO_2_

**Fig. 5:**
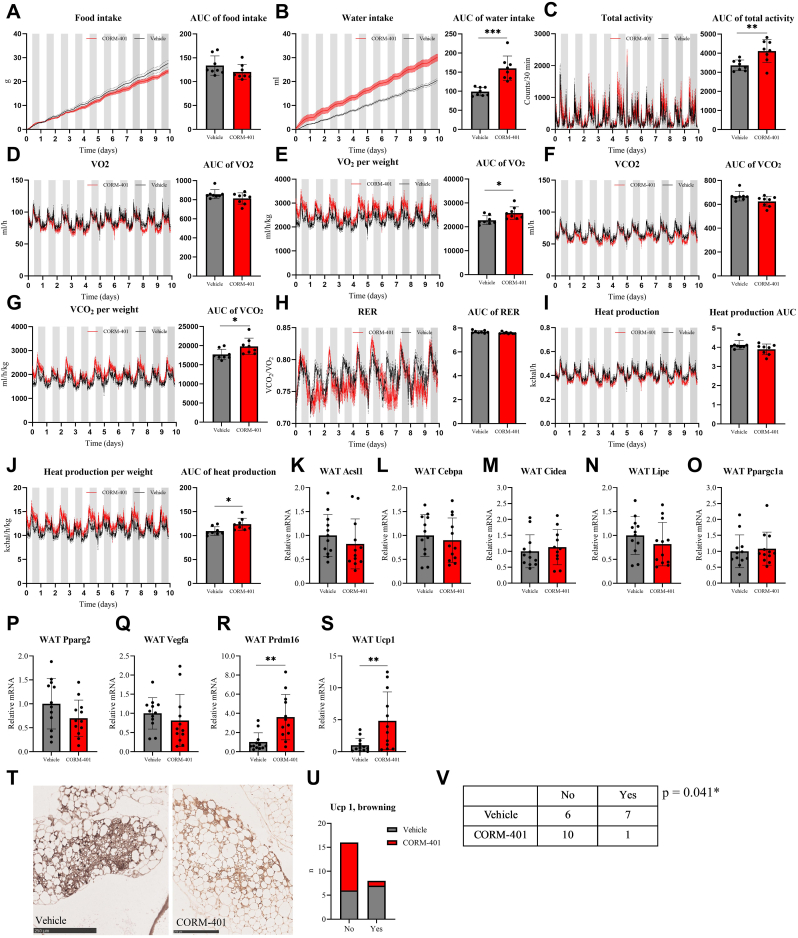
Home cage analysis and analysis of WAT browning. *n* = 9-12 in each group. Data from measurements are shown in plots accompanied by the associated area under the curve values (AUC). All AUC values and plots are shown as mean ± SD. A, food intake. B, water intake. C, total activity. D, oxygen (O_2_) consumption. E, O_2_ consumption related to mass. F, carbon dioxide (CO_2_) production. G, CO_2_ production related to mass. H, respiratory exchange ratio (RER). I, heat production. J, heat production relative to mass. K–S, qPCR analysis of WAT mRNA levels of CORM-401-treated mice relative to vehicle, studied relative to *Ppia* mRNA. T, immunohistochemical analysis of UCP1 staining of WAT. U, browning of WAT based on UCP1 immunostaining. V, Fisher's exact of browning of WAT. *Ascl1*, achaete-scute family bHLH transcription factor 1; *Cebpa*, CCAAT/enhancer-binding protein a; *Cidea*, cell death-inducing DFFA-like effector a; *Lipe*, hormone-sensitive lipase; *Ppargc1a*, peroxisome proliferator-activated receptor gamma coactivator 1a; *Pparg2*, peroxisome proliferator-activated receptor gamma 2; *Prdm16*, pr domain-containing 16; *Ucp1*, uncoupling protein1; *Vegfa*, vascular endothelial growth factor a. ∗*p* < 0.05, ∗∗*p* < 0.01.

To measure drinking and feeding behavior, locomotor activity and breathing gases under a long-term CORM-401 *vs.* vehicle treatment, mice were taken to home cage analysis after 6 weeks of treatment. No difference in food intake during the 10-day monitoring period was observed between the CORM-401 and vehicle-treated groups (Fig. 5A). Water intake, however, was drastically 60% greater in the CORM-401-treated mice, the difference increasing steadily throughout the 10-day monitoring period (Fig. 5B). Acute CO poisoning causes dizziness and even loss of consciousness. Althoug the COHb levels of the CORM-401-treated mice were clearly below poisonous, these mice were much more active than the vehicle-treated mice (Fig. 5C). Additionally, the CORM-401-treated mice had a subtle increase in oxygen consumption per body weight but they also produced slightly more carbon dioxide per body weight, suggesting a modest increase in metabolic rate (Fig. 5D–G). No statistically significant difference was observed in respiratory exchange rate (RER) between the treatment groups but the CORM-401-treated mice showed a higher RER variability compared to vehicle-treated mice (Fig. 5H) which is in line with an increased activity level. CORM-401-treated mice produced more heat per body weight compared to the vehicle treated mice (Fig. 5I and J).

As the CORM-401-treated mice produced more heat and drank more water than their vehicle-treated counterparts, we next investigated WAT browning as a potential mechansim by examining the expression of browning-associated mRNAs in WAT and the histology of the WAT. No significant difference in achaete-scute family bHLH transcription factor 1 (*Ascl1*), CCAAT/enhancer-binding protein a (*Cebpa*), cell death-inducing DFFA-like effector a (*Cidea*), hormone-sensitive lipase (*Lipe*), peroxisome proliferator-activated receptor gamma coactivator 1α (*Ppargc1a*), peroxisome proliferator-activated receptor gamma 2 (*Pparg2*) or vascular endothelial growth factor a (*Vegfa*) relative mRNA levels were observed between the treatment groups (Fig. 5K–Q). However, relative mRNA levels of pr domain-containing 16 (*Prdm16*) and uncoupling protein 1 (*Ucp1*) were significantly upregulated in the CORM-401-treated WAT compared to vehicle (Fig. 5R and S). Despite of the increase in mRNA levels, when assessed by immunohistochemistry the overall UCP1 expression was more pronounced in the vehicle than CORM-401-treated WAT (Fig. 5T–V).

### 3.6 CORM-401 causes dehydration and lactatemia-induced metabolic acidosis which is compensated through increased respiration

**Fig. 6:**
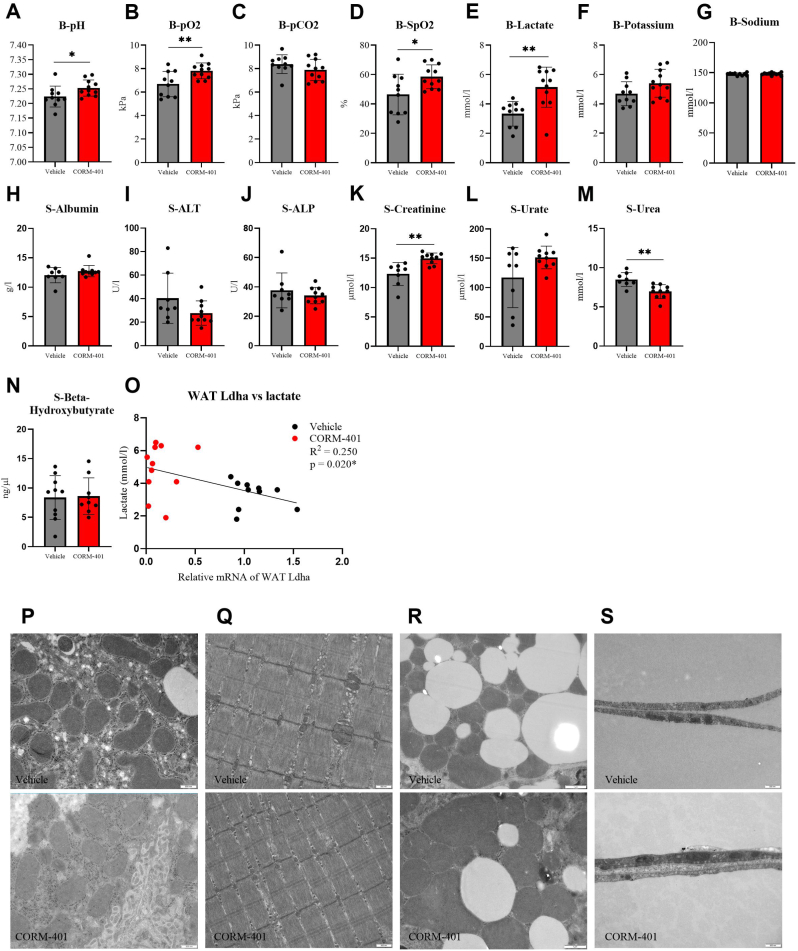
Safety analyses of long-term CORM-401 treatment. *n* = 9-12 in each group. A, pH. B, partial pressure of oxygen (pO_2_). C, partial pressure of carbon dioxide (pCO_2_). D, peripheral oxygen saturation (SpO_2_). E, lactate. F, Potassium (P), G, sodium (Na). H, serum albumin. I, serum alanine aminotransferase (ALT). J, serum alkaline phosphatase (ALP). K, serum creatinine. L, serum urate. M, serum urea. N, serum β-hydroxybuturate. O, association between blood lactate and WAT lactate dehydrogenase (*Ldha*) relative mRNA levels. P–S, representative images of a transmission electron microscope (TEM) imaging; P, liver; Q, skeletal muscle; R, BAT and S, WAT. ∗*p* < 0.05, ∗∗*p* < 0.01.

To assess safety of long-term CORM-401 treatment, we conducted venous blood gas analysis and evaluated organ damage-related metabolic measures from serum. The CORM-401-treated mice had higher pH levels being slightly more alkalotic, and they had higher partial oxygen pressure (pO_2_), peripheral oxygen saturation (SpO_2_) and lactate levels, with a trend also observed for a lower partial pressure of carbon dioxide (pCO_2_) (Fig. 6A–E), consistent with mild metabolic acidosis induced by lactatemia, and compensated through increased respiration. The CORM-401-treated mice also showed a trend for slightly higher levels of potassium (P) compared to their vehicle-treated counterparts whereas no difference in sodium levels was detected (Fig. 6F and G).

Of the serum measures, albumin, ALT and ALP levels were similar in the treatment groups whereas serum creatinine levels were higher in CORM-401-treated mice (Fig. 6H–J). As both potassium and creatinine levels were higher in the CORM-401-treated mice, we further examined serum urate and urea levels, and renal histology finding lower urea levels in the CORM-401-treated serum whereas no other differences were observed between the experimental groups, suggesting possible dehydration of the CORM-401-treated mice but similar kidney function, supported also by no difference in the calculated serum osmolality (326 ± 6 *vs.* 324 ± 4 mmol/l for vehcile *vs.* CORM-401, p = 0.46, respectively), in both groups (Fig. 6K–M, S3A and S3B). To investigate potential increase in ketone bodies, serum beta-hydroxybutyrate levels were measured but no differences were observed between the treatment groups (Fig. 6N).

To evaluate potential causes of the lactatemia, we next evaluated associations of lactate levels and other variables. The association between blood COHb and lactate levels was minor (*R*^*2*^ = 0.038, p = 0.039) whereas a more pronounced negative association between relative mRNA level of WAT *Ldha* and blood lactate levels was observed, potentially suggesting a negative feedback (Fig. 6O).

To examine potential organ damage, we further examined tissue ultrastructure by transmission electron microscopy (TEM) focusing especially on mitochondria, which in earlier studies have been identified as the main target of CORM-401 [24]. No significant differences were observed in the liver, skeletal muscle, WAT, or BAT between the CORM-401 and vehicle-treated groups (Fig. 6P–S). Examination of the skeletal muscle ultrastructure revealed a regular and well-organized architecture that did not differ between groups, suggesting absence of muscle damage in the CORM-401-treated mice (Fig. 6Q). We also assessed mitochondrial respiratory chain by measuring cytochrome *c* oxidase subunit 1 (*Cox1*) mRNA expression relative to nuclear encoded TATA-box binding protein (*Tbp*), and found no differences between CORM-401-treated and vehicle groups in WAT or BAT (Fig. S3C and S3D).

### 3.7 Potential mechanisms underlying the elevated cholesterol levels and the observed weight loss

**Fig. 7:**
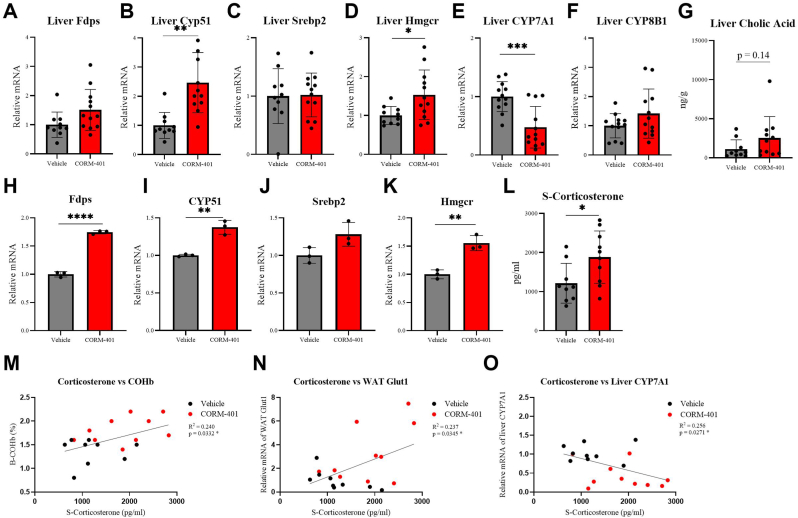
Cholesterol and bile acid metabolism and corticosterone levels. *n* = 9-12 in each group. A-F, relative mRNA levels of key cholesterol and bile acid synthesis genes in liver. G, liver cholic acid concentration. H–K, relative mRNA levels of key cholesterol synthesis genes in primary mouse hepatocytes. L, serum corticosterone concentration. M, association between corticosterone and COHb. N, association between corticosterone and relative mRNA levels of WAT *Glut1*. O, association between corticosterone and liver *Cyp7a1* relative mRNA levels. *Fdps*, farnesyl diphosphate synthase; *Cyp51*, lanesterol 14-α-demethylase; *Srebp2*, sterol regulatory element-binding protein 2; *Hmgcr*, 3-hydroxy-3-methylglutaryl-CoA reductase; *Cyp7a1*, cholesterol 7α-hydroxylase; *Cyp8b1*, sterol 12α-hydroxylase. ∗*p* < 0.05, ∗∗*p* < 0.01, and ∗∗∗*p* < 0.001.

Given the substantial decrease in WAT mass and the simultaneous increase in serum total and LDL + VLDL cholesterol levels following the 7-week CORM-401 treatment, we proceeded to examine hepatic cholesterol and bile acid metabolism more comprehensively. In the liver, lanesterol 14-α-demethylase (*Cyp51*) and 3-hydroxy-3-methylglutaryl-CoA reductase (*Hmgcr*) mRNA levels were upregulated in the CORM-401-treated mice suggesting activation of the cholesterol synthesis whereas no significant difference in farnesyl diphosphate synthase (*Fdps*) and sterol regulatory element-binding protein 2 (*Srebp2*) mRNA levels were seen between the treatment groups (Fig. 7A–D). Statistically significant downregulation was observed in the key hepatic bile acid metabolism enzyme cholesterol 7α-hydroxylase (*Cyp7a1*) mRNA in the CORM-401-treated liver when compared to vehicle but no difference was seen in sterol 8α-hydroxylase (*Cyp8b1*) mRNA levels (Fig. 7E–F). There was no significant difference in liver cholic acid concentration between the treatment groups (Fig. 7G). In order to study whether the effect of CORM-401 on cholesterol metabolism was direct, we treated primary mouse hepatocytes with it for 24 h and found significant upregulation of *Cyp51*, *Hmrcr* and *Fdps* mRNA but not *Srebp2* mRNA compared to vehicle treatment (Fig. 7H–K). Together, these data could suggest an upregulation of hepatic cholesterol synthesis and downregulation of bile acid synthesis following CORM-401-treatment.

As the above findings did not offer a direct explanation for the increased WAT lipolysis, we next measured serum corticosterone levels which are known to stimulate adipose tissue lipolysis in rodents. The CORM-401-treated mice exhibited a pronounced 1.6 x elevation in corticosterone levels, suggesting a prolonged stress reaction (Fig. 7L). A positive association was observed between serum corticosterone levels and blood COHb levels (Fig. 7M). There was also a positive association between serum corticosterone levels and relative mRNA of WAT *Glut1*, and a negative association between serum corticosterone levels and relative mRNA of hepatic *Cyp7a1* (Fig. 7N and O), suggesting an association between the elevated systemic glucocorticoid levels and the metabolic alterations in tissues.

## 4 Discussion

The aim of this study was to evaluate the metabolic effects of long-term CORM-401 treatment in an obese mouse model, and analyze whether these effects were mediated by the HIF pathway. The study further included an examination of the underlying mechanisms of action of the CORM-401 molecule. Here, the observed marked reduction in WAT mass and total body mass following CORM-401 treatment prompted a deeper examination of lipid metabolism. In previous studies, CORM-401-treated mice exhibited attenuated weight gain compared with controls, but actual weight loss has not been reported to the best of our knowledge [24,25]. Our data strongly point toward stress-induced lipolysis as the principal driver of the metabolic phenotype including lowered WAT inflammation and elevated serum VLDL + LDL cholesterol levels. CORM-401-treated mice exhibited a pronounced elevation in the levels of circulating corticosterone, a glucocorticoid hormone hallmark of systemic stress activation and a potent stimulator of lipid mobilization. A positive association between serum corticosterone levels and blood COHb levels was observed.

Elevated circulating corticosterone levels in mice were associated with repression of hepatic *Cyp7a1* mRNA, while *Cyp8b1* mRNA expression and hepatic cholic acid levels remained unchanged. This pattern is consistent with a glucocorticoid-mediated activation of the hepatic glucocorticoid receptor, which enhances enterohepatic bile acid recycling and activates FXR-dependent feedback pathways, leading to rapid suppression of *Cyp7a1* without immediate depletion of the bile acid pool [32,33]. In contrast, *Cyp8b1* and cholic acid content, which are strongly buffered by the enterohepatic circulation and gallbladder storage, are less sensitive to early feedback regulation [34,35]. Thus, these findings likely reflect an adaptive feedback state in which bile acid synthesis is transcriptionally restrained while systemic bile acid homeostasis is preserved.

Several physiological and behavioral observations were consistent with activation of the stress response in the CORM-401-treated mice. Despite a drastic difference in body weight, the clearly leaner CORM-401-treated mice were only slightly more insulin sensitive than their heavier vehicle-treated counterparts, which could stem from glucocorticoid-induced hyperglycemia. Food intake did not differ significantly between the treatment groups, yet CORM-401-treated mice displayed markedly increased water consumption and a substantially higher locomotor activity; features opposite to the reduced activity typically associated with CO toxicity. These mice also exhibited increased oxygen consumption, and higher carbon dioxide output, indicating an overall rise in metabolic rate with a higher variability in respiratory exchange ratio, and possibly hyperventilation. Some of these findings are consistent with previous observations reported for another CO-releasing molecule; CORM-A1-treatment in HFD-fed mice, where its chronic administration increased oxygen consumption and heat production compared with inactive CORM-A1-treated controls, and attenuated development of obesity and reduced body fat and fasting glucose and insulin levels but did not diminish AUC of GTT [36]. In addition, CORM-401-treated mice showed elevated serum creatinine levels without evidence of renal injury, suggesting relative dehydration. The dehydration may have contributed to the observed body weight differences between groups and could also partly explain the increased water intake in CORM-401 treated mice. Furthermore, in the context of the enhanced lipolytic response observed in CORM-401 treated mice, it is noteworthy that each lipase involved in the sequential hydrolysis of triacylglycerol to diacylglycerol and monoacylglycerol is a hydrolase that consumes water. Increased lipolytic flux may therefore further exacerbate dehydration, reinforcing the physiological picture observed in these animals [37]. Furthermore, the observed stress response of the CORM-treated mice, evidenced by elevated corticosterone levels, could have plausibly contributed to dehydration.

The CORM-401-treated mice also showed elevated heat production. Increased heat production by CORM-401 has previously been reported in studies conducted in rats [38]. Despite earlier reports [38], our analysis did not support an enhanced thermogenic activation as the major underlying cause of the increased energy expenditure in CORM-401-treated mice. Although WAT *Prdm16* and *Ucp1* mRNA levels were upregulated in CORM-401-treated mice, histological browning was more pronounced in the vehicle-treated WAT, perhaps mediated by the HFD feeding. Moreover, the BAT mass was reduced by 45% in the CORM-401-treated mice. Together, these findings suggest that the metabolic state induced by CORM-401 is better explained by stress hormone-mediated alterations rather than classical CO toxicity, or BAT or WAT browing-driven increased thermogenesis. Previous studies have shown that CO can promote mitochondrial respiration, mitochondrial biogenesis, and cytoprotective metabolic adaptation, potentially through transient and partial inhibition of cytochrome *c* oxidase (complex IV), mild uncoupling [39]. Therefore, in addition to respiratory compensation to metabolic acidosis, it is plausible that some of the metabolic effects observed after CORM-401 treatment reflect a CO-mediated adaptive enhancement of mitochondrial function.Despite of CORM-401 showing direct inhibitory potential towards all three HIF–P4Hs, robust stabilization of HIFα was not observed across tissues of the CORM-401-treated mice. Direct HIF1α stabilization was only detectable in 25% of the studied samples in WAT and liver, suggesting that activation of the canonical hypoxia response was mild under the dosing regimen used. However, detection of HIFα in tissue samples is known to be notoriously challenging which may have contributed to low detection. Nevertheless, the transcriptional profile of the CORM-401-treated mouse group revealed changes consistent with HIF-mediated metabolic reprogramming. In WAT, *Glut1* mRNA, a well-established HIF target gene, was significantly upregulated, while in skeletal muscle expression of *Glut4* mRNA was increased, both aligning with an enhanced glucose uptake typically associated with increased HIF signaling [[9], [10], [11]]. However, the absence of detectable HIFα stabilization in the CORM-401-treated skeletal muscle leaves open the possibility that upregulation of *Glut4* mRNA was due to another mediator, for example AMPK or mitochondrial stress signaling [40]. Hepatic upregulation of *Pdk1* mRNA, another classical HIF target, and higher blood lactate levels further support the presence of low-grade HIF pathway engagement. Whether these changes were mediated by direct inhibition of HIF–P4Hs 1-3 by the released CO, suggested to compete with binding of O_2_ [21], or potentially via local hypoxia caused by elevated COHb levels, cannot be answered with these data. Estimated tissue concentration of CORM-401 when administered at 30 mg/kg in a 34 g mouse, the average starting weight, if distributed in total body water is about 130 μM which is in line with the here determined IC50 values of CORM-401 for HIF–P4Hs 1-3; 150-320 μM. CORM-401 releases on average 3.2 mol equivalents of biologically active CO efficiently and rapidly (half-life 0.8 min) when a suitable capturer, *e.g.* myoglobin, is available [41]. Considering the role of liver in metabolsim of orally administered xenobiotics and the fact that CORM-401 has been reported to accumulate in adipose tissue [24], the concentrations in these tissues are likely higher. The IC50 values are also in the same range with the oxygen *K*_*m*_ values for HIF–P4Hs (230-250 μM) [30], supporting the inhibition in the *in vitro* enzymatic assay being mediated by competition of CO with oxygen binding.

## 5 Strengths and limitations

This study has several notable strengths, including its comprehensive multi-tissue metabolic characterization, integration of endocrine, behavioral, and physiological data, and systematic evaluation of the alternative mechanisms such as classical CO toxicity, mitochondrial uncoupling, and thermogenic browning. The analyses rule out tissue damage while identifying a dual mechanism; glucocorticoid-driven stress activation accompanied by a low-grade, tissue specific HIF engagement, further strengthening the mechanistic interpretation.

However, the study also has limitations. Although corticosterone levels were elevated, the study does not include study of upstream markers *e.g.*, ACTH, CRH or adrenal histology due to lack of tissue samples. Dehydration is suggested by elevated creatinine levels, increased water intake and lipolysis-associated water consumption but measures of plasma osmolality, hematocrit or urine concentration were not performed, limiting the dehydration hypothesis. Increased locomotor activity may confound interpretations of the energy expenditure, whether hyperactivity is a cause or consequence of the stress response.

## 6 Conclusions

Together, these findings identify a glucocorticoid-mediated stress activation as a driver of the metabolic effects of CORM-401, with low-grade HIF pathway engagement acting as a potential secondary contributor. This model of dual-mechanism provides new insight into how CO-releasing molecules influence whole-body metabolism and highlights the importance of considering stress physiology when evaluating their therapeutic potential.

## CRediT authorship contribution statement

**Emma Klemola:** Data curation, Formal analysis, Investigation, Visualization, Writing – original draft. **Karoliina Posio:** Formal analysis, Investigation, Visualization. **Mikko Karpale:** Investigation. **Elitsa Y. Dimova:** Formal analysis, Investigation. **Ghulam S. Raza:** Investigation. **Kari A. Mäkelä:** Investigation. **Irina I. Nagy:** Resources. **Ilkka Miinalainen:** Investigation. **Joona Tapio:** Conceptualization, Data curation, Formal analysis, Investigation, Supervision, Writing – review & editing. **Peppi Koivunen:** Conceptualization, Data curation, Funding acquisition, Project administration, Resources, Supervision, Writing – original draft, Writing – review & editing.

## Declaration of competing interest

The authors declare the following financial interests/personal relationships which may be considered as potential competing interests: Peppi Koivunen reports financial support was provided by Research Council of Finland, The Jane and Aatos Erkko Foundation, and Sigrid Jusélius Foundation. Other authors declare that they have no known competing financial interests or personal relationships that could have appeared to influence the work reported in this paper.

## Data Availability

Data will be made available on request.
