# Supplementary material for: Carbon monoxide-releasing molecule CORM-401 treatment elicits corticosterone-driven stress lipolysis and tissue-specific hypoxia-inducible factor activation
Source: Redox Biol. 2026 Jul 10;95:104295. doi: 10.1016/j.redox.2026.104295 (PMC13382091; doi:10.1016/j.redox.2026.104295)
Supplement: Multimedia component 1 [file mmc1.pdf]

Supplementary data

Carbon monoxide-releasing molecule CORM-401 treatment elicits corticosterone-driven stress lipolysis and tissue-specific hypoxia-inducible factor activation

Emma Klemola[1,2], Karoliina Posio[1,2], Mikko Karpale[1,2], Elitsa Y. Dimova[1,2], Ghulam S. Raza[2,3], Kari A. Mäkelä[2,3], Irina Nagy[4], Ilkka Miinalainen[2], Joona Tapio[1,2], and Peppi Koivunen[1,2*]

[1]Research Unit of ECM & Hypoxia, Faculty of Medical Biochemistry and Molecular Medicine, University of Oulu, Finland

[2]Biocenter Oulu, University of Oulu, Finland

[3]Research Unit of Biomedicine and Internal Medicine, Faculty of Medicine, University of Oulu, Oulu, Finland

[4]Department of Clinical Chemistry, Cancer and Translational Medicine Research Unit, Medical Research Center, University of Oulu and Northern Finland Laboratory Centre NordLab, Oulu University Hospital, Oulu, Finland

*Correspondence: peppi.karppinen@oulu.fi

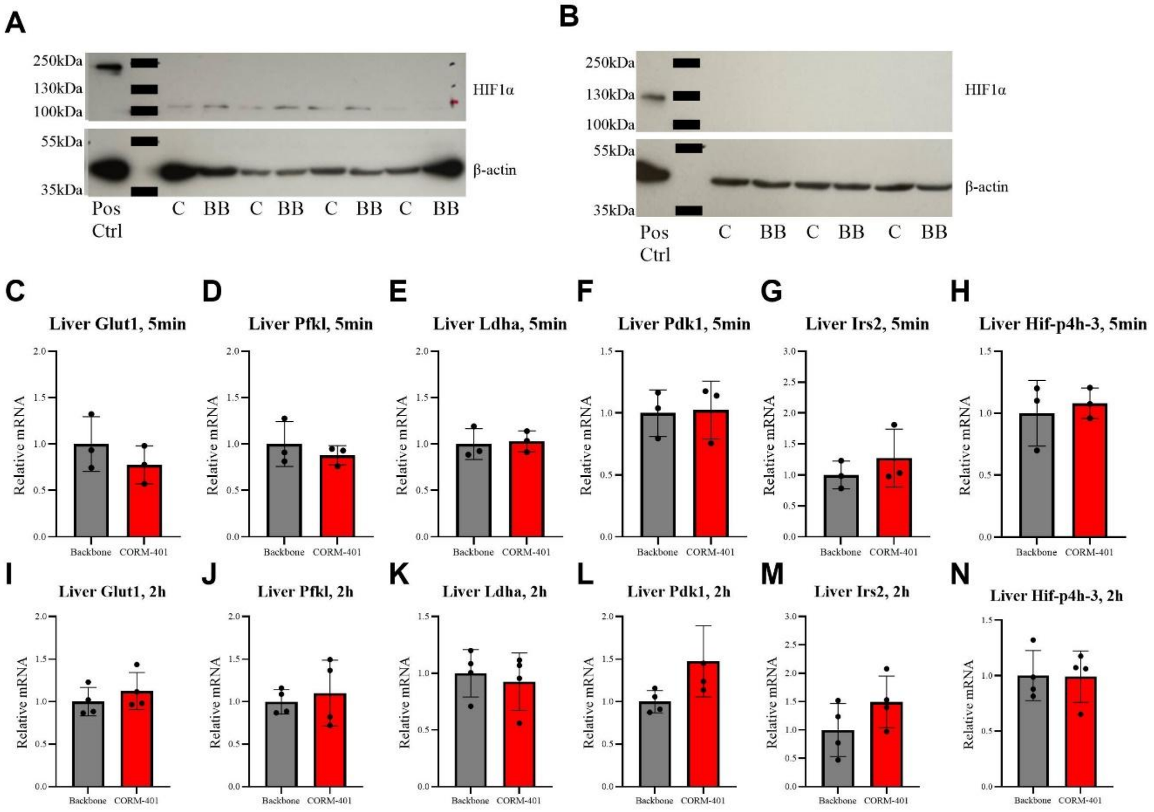

Figure S1. Single dose pilot experiment of CORM-401. A-B, HIF1α western blot of liver at 5 min and at 2 h timepoint, respectively. C-H, qPCR analysis of liver mRNA levels of CORM-401-treated mice (n = 3) relative to vehicle (n = 3) at 5 min timepoint, studied relative to *B2m* mRNA. I-N, qPCR analysis of liver mRNA levels of CORM-401-treated mice(n = 4) relative to vehicle (n = 4) at 2 h timepoint, studied relative to *B2m* mRNA. C, CORM-401; BB, backbone; Pos Ctr, positive control. *Glut1*, glucose transporter 1; *Pfkl,* phosphofructokinase liver type; *Ldha*, lactate dehydrogenase a; *Pdk1*, pyruvate dehydrogenase kinase 1; *Irs2*, insulin receptor substrat2; *Hif-p4h-3*, hypoxia-inducible factor prolyl-4-hydroxylase 3.

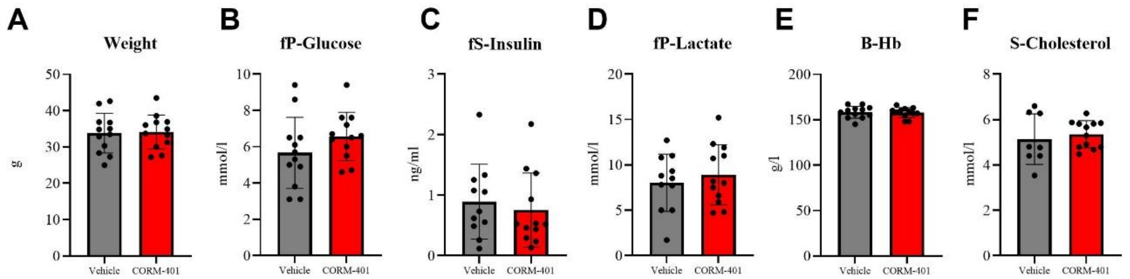

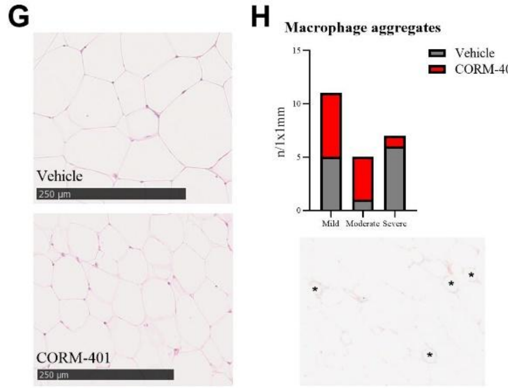

I

	Mild	Moderate	Severe
Vehicle	5	1	6
CORM-401	6	4	1

p = 0.041*

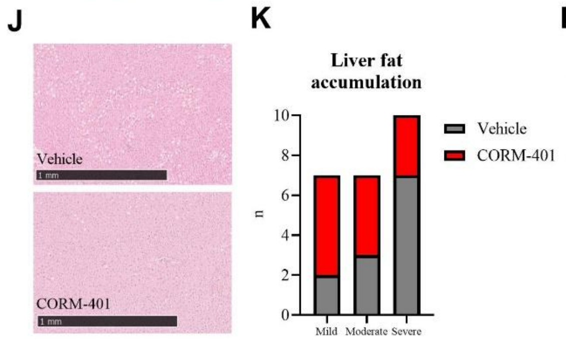

L

	Mild	Moderate	Severe
Vehicle	2	3	7
CORM-401	5	4	3

p = 0.047*

Figure S2. Baseline measurements and histological analysis of WAT and liver at sacrifice. *n* = 9-12 in each group. A, weight at baseline. B, fasting glucose at baseline. C, fasting insulin at baseline. D, fasting lactate at baseline. E, hemoglobin (Hb) at baseline. F, serum cholesterol concentration at baseline. G, Hematoxylin and eosin (HE) – staining of WAT. H, macrophage aggregate accumulation and representative CD68 – staining of WAT, aggregates indicated in asterisk. I, Fisher's exact of macrophage aggregates. J, Hematoxylin and eosin (HE) – staining of liver. K, liver fat accumulation. L, Fisher's exact of liver fat accumulation.

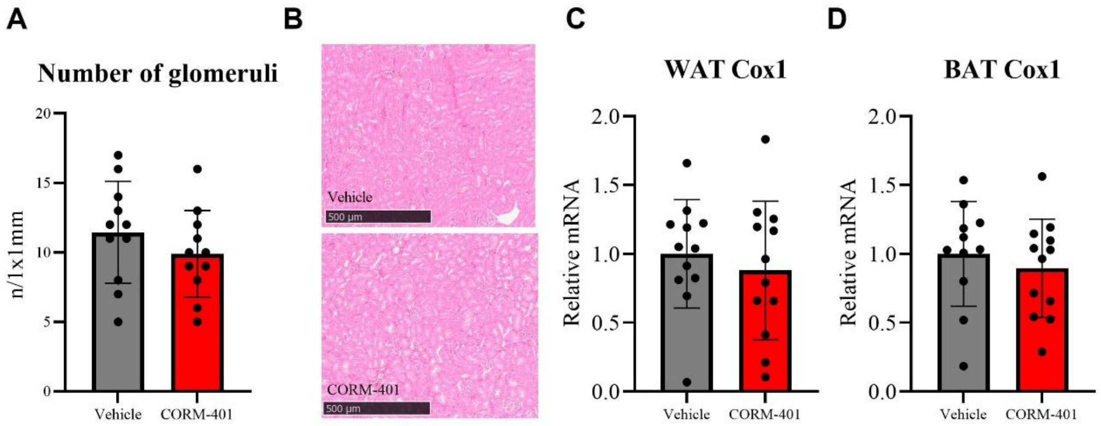

Figure S3. Histological analyses of kidney and expression of mitochondrial mRNA in WAT and BAT. n = 9-12 in each group. A, number of glomeruli. B, hematoxylin and eosin (HE) staining of kidney. C, Relative mRNA of *Cox1* in WAT studied relative to *Tbp*. D, relative mRNA of *Cox1* in BAT studied relative to *Tbp*. *Cox1*, cytochrome c oxidase subunit I; *Tbp*, TATA-box binding protein.
